# High serum prevalence of autoreactive IgG antibodies against peripheral nerve structures in patients with neurological post-COVID-19 vaccination syndrome

**DOI:** 10.3389/fimmu.2024.1404800

**Published:** 2024-08-02

**Authors:** Friederike A. Arlt, Ameli Breuer, Elli Trampenau, Fabian Boesl, Marieluise Kirchner, Philipp Mertins, Elisa Sánchez-Sendín, Mahoor Nasouti, Marie Mayrhofer, Martin Blüthner, Matthias Endres, Harald Prüss, Christiana Franke

**Affiliations:** ^1^ Department of Neurology and Experimental Neurology, Charité-Universitätsmedizin Berlin, corporate member of Freie Universität and Humboldt-Universität zu Berlin, Berlin, Germany; ^2^ German Center for Neurodegenerative Diseases (DZNE) Berlin, Berlin, Germany; ^3^ Core Unit Proteomics, Berlin Institute of Health at Charité – Universitätsmedizin Berlin and Max Delbrück Center for Molecular Medicine, Berlin, Germany; ^4^ Department of Autoimmune Diagnostics, Medizinisches Versorgungszentrum (MVZ) Laboratory PD Dr. Volkmann & Colleagues, Karlsruhe, Germany; ^5^ German Center for Cardiovascular Diseases (DZHK), Berlin, Germany; ^6^ Center for Stroke Research Berlin (CSB), Charité-Universitätsmedizin Berlin, corporate member of Freie Universität and Humboldt-Universität zu Berlin, Berlin, Germany; ^7^ German Center for Mental Health (DZPG), Berlin, Germany

**Keywords:** SARS-CoV-2 vaccination, COVID-19 vaccination, post-COVID-19 vaccination syndrome (PCVS), autoantibody, peripheral nerve, neurofilament autoantibodies, DFS-70

## Abstract

**Background:**

Patients suffering from neurological symptoms after COVID-19 vaccination (post-COVID-19 vaccination syndrome (PCVS)) have imposed an increasing challenge on medical practice, as diagnostic precision and therapeutic options are lacking. Underlying autoimmune dysfunctions, including autoantibodies, have been discussed in neurological disorders after SARS-CoV-2 infection and vaccination. Here, we describe the frequency and targets of autoantibodies against peripheral nervous system tissues in PCVS.

**Methods:**

Sera from 50 PCVS patients with peripheral neurological symptoms after COVID-19 vaccination and 35 vaccinated healthy controls were used in this study. IgG autoreactivity was measured via indirect immunofluorescence assays on mouse sciatic nerve teased fibers. The frequencies of autoantibodies were compared between groups using Fisher’s exact test. Serum anti-ganglioside antibodies were measured in ganglioside blots. Autoantibody target identification was performed using immunoprecipitation coupled to mass spectrometry. Subsequent target confirmation was conducted via cell-based assays and ELISA.

**Results:**

Compared with controls, PCVS patients had a significantly greater frequency of autoantibodies against peripheral nervous system structures (9/50(18%) vs 1/35(3%); p=0.04). Autoantibodies bound to paranodes (n=5), axons (n=4), Schmidt-Lanterman incisures (n=2) and Schwann cell nuclei (n=1). Conversely, antibodies against gangliosides were absent in PCVS patients. Target identification and subsequent confirmation revealed various subunits of neurofilaments as well as DFS-70 as autoantibody epitopes.

**Conclusion:**

Our data suggest that autoantibodies against nervous system tissue could be relevant in PCVS patients. Autoantibodies against neurofilaments and cell nuclei with so far non-established links to this disease spectrum should be further elucidated to determine their biomarker potential.

## Introduction

Vaccination development has succeeded in fighting the pandemic caused by severe acute respiratory syndrome coronavirus 2 (SARS-CoV-2), and several vaccines have been shown to harbor only mild and transient side effects (1–4). However, similar to post-COVID-19 syndrome (PCS), which represents a spectrum of neurological symptoms that occur after SARS-CoV-2 infection (C. 5–7), various persistent neurological symptoms following COVID-19 vaccination have been reported and can be referred to as post-COVID-19 vaccination syndrome (PCVS) (8, 9).

In contrast to PCS patients, who predominantly report central nervous system symptoms such as fatigue or cognitive deficits (5), PCVS patients predominantly report peripheral nervous system symptoms such as paresthesia and neuropathic pain (9). Due to the lack of definition of the syndrome and the absence of pathological findings in routine diagnostic methods, including electrophysiological examinations, the diagnosis of PCVS remains difficult to distinguish from other diseases, including somatic symptom disorders (9).

Although rare compared to the total number of vaccines administered, autoimmune phenomena have been reported in association with SARS-CoV-2 vaccination (10). In adverse events, the underlying pathophysiological mechanism was vaccine-dependent induction of pathologic autoreactive antibodies (11). However, the contribution of autoantibodies to PCVS symptoms, particularly after mRNA-based vaccination, has not been determined. We therefore aimed to analyze the frequency and epitopes of autoreactive antibodies against structures of the peripheral nerve in a cohort of 50 PCVS patients.

## Materials and methods

### Patient population and clinical and laboratory diagnostics

Study participants were referred to our neurology outpatient clinic at Charité - Universitätsmedizin Berlin between October 2021 and July 2022 when neurological symptoms arose in temporal relation to the SARS-CoV-2 vaccination. Patients had to receive at least one COVID-19 vaccination and to report new-onset symptoms within one month after vaccination. Patients were excluded if they had a confirmed SARS-CoV-2 infection prior to symptom onset or if an alternative condition related to their symptoms had been diagnosed. The results of the comprehensive clinical and laboratory analyses as part of our standard assessment have been previously published. (9) This included standard electrophysiological examination and, if normal, skin biopsies investigating small fiber pathology in patients complaining of paresthesia and neuropathic pain. Laboratory workup was performed following the guidelines of the German Neurological Society for the diagnosis of polyneuropathy. In this study, we further conducted autoantibody diagnostics, including screening for antinuclear antibodies (ANA) (Hep-2 indirect immunofluorescence, EUROIMMUN), antibodies against extractable nuclear antigens (Ro/SS-A, La/SS-B, RNP/Sm, Sm, SCL-70, Centromer-B, Jo-1) (ELISA, EUROIMMUN), anti-neutrophil cytoplasmic antibodies (cANCA and pANCA) (ELISA, EUROIMMUN), and antibodies against Mi-2-alpha and -beta, TIF1g, MDA5, NXP2, Ku-80, PM-Scl 100/75, SRP, Jo-1, PL-7, PL-12, EJ, OJ, SAE and Ro-52 (Immunoblot, EUROIMMUN), strictly following the manufacturer’s instructions. 35 age- and sex-matched vaccinated healthy health care workers were used as controls.

### Tissue-based immunofluorescence assays

Sciatic nerve dissection from wild-type mice and further processing were performed as previously described (12). Briefly, nerves were dissected and directly fixed in 4% paraformaldehyde (PFA) for 20 min on ice. After removal of the epineurium, the nerves were teased on glass slides, air-dried overnight, and stored at -20°C. Prior to staining, the teased fibers were postfixed and permeabilized with 100% methanol for 2 min at -20°C and washed with phosphate-buffered saline (PBS). Postfixed nerves were blocked in blocking solution (10% normal goat serum, 2.5% bovine serum albumin and 0.1% Triton-X) and stained with patient and control sera at a dilution of 1:500 in blocking solution at 4°C overnight. Additionally, a purified anti-neurofilament heavy chain (NF-H) antibody (#801702, Biolegend, San Diego, CA, USA; final dilution 1:200) was used. After washing, secondary antibodies against human IgG conjugated to Alexa488 (#109–545-003, Dianova, Hamburg, Germany; final dilution 1:1,000) and Alexa594-labeled goat anti-mouse IgG (#115–585-03, Jackson Research, Baltimore, PA, USA; final dilution 1:500) were applied for 1 h at room temperature. Cell nuclei were stained with DAPI. All sera were stained in two technical replicates.

### Ganglioside blots

Autoantibodies against gangliosides were analyzed using a linear elimination (LINE) immunoassay (GA Generic Assays GmbH) according to the manufacturer’s instructions. The immunoassay mixture included membranes coated with 11 different gangliosides (GM1, GM2, GM3, GM4, GD1a, GD1b, GD2, GD3, GT1a, GT1b and GQ1b) and sulphatides. Briefly, the serum samples stored at -20°C were carefully shaken after thawing to ensure homogeneity. The sera were diluted 1:100 and incubated for 2 h at 4°C to allow sufficient binding of the autoantibodies to the gangliosides immobilized on the solid phase. Unbound sample components were removed by a washing step. The bound autoantibodies reacted specifically with anti-IgG or anti-IgM conjugated with horseradish peroxidase during a second incubation step of 60 min at 4°C. Excess conjugate was removed by an additional washing step. After the addition of substrate (tetramethyl benzidine) and incubation for 10 min at room temperature, the strips were dried for 30 min and read with a scanner. Samples were considered positive if the intensity of the respective test line was higher than the cutoff defined in the software.

### Immunoprecipitation coupled to mass spectrometry (IP-MS)

Nervous system tissue of wild-type mice was used as IP input. The tissue was cryo-grinded, solubilized in lysis buffer, and cleared using 18,000 rpm centrifugation for 30 min at 4°C. Meanwhile, the serum samples were coupled to Dynabeads™ Protein G (#10004D, Invitrogen by Thermo Fisher Scientific, Vilnius, Lithuania) in 50 mM hydroxyethyl-piperazineethanesulfonic acid (HEPES) for 30 min. After washing, the complexes of beads and antibodies were incubated with the cleared lysates for 1 h at 4°C. After washing with wash buffer containing 300 mM sodium chloride (NaCl) in 50 mM HEPES and 0.05% Tween-20, the antibody-dynabead complexes were subjected to on-bead digestion with LysC and trypsin and subsequent mass spectrometry-based proteomic analyses via liquid chromatography tandem mass spectrometry (LC−MS/MS). Briefly, the peptides were separated on a reversed-phase C18 column for 90 min on a high-performance liquid chromatography (HPLC) system (Thermo Fisher Scientific) and analyzed on a Q Exactive Plus instrument (Thermo Fisher Scientific) in data-dependent mode. The raw data were analyzed with the MaxQuant software package using the human and mouse UniProt databases (HUMAN.2020–06; MOUSE.2019–07) and an FDR of 1% for peptide and protein identification. LFQ (label-free quantitation) intensities were used for statistical analyses. Antibody targets were identified by performing group comparisons against the negative control using Student´s t-test and a significance cutoff of false discovery rate (FDR) 5%. Solely human proteins were excluded from the analysis as potential contaminants originating from serum.

### Cell-based immunofluorescence assays

Cell-based testing of IgG reactivity against neuronal intermediate filament antigens was adapted from previously published protocols (13). In brief, commercially available plasmids encoding Neurofilament heavy chain (NFH; #RC213487 Origene, Rockville, MD, USA), Neurofilament medium chain (NFM; #RC224475 Origene), Neurofilament light chain (NFL; #RC205920 Origene), Peripherin (PRPH; #RC207561 Origene), and Alpha-Internexin (INA; #RC202877 Origene) were transiently expressed in HEK293 cells using PEI-mediated transfection on polylysine-coated coverslips. After 48 h, the cells were fixed and permeabilized with 100% methanol for 3 min at -20°C and washed with PBS. After blocking, the cells were incubated with the serum of patient #12 (dilution of 1:600) and an anti-Myc monoclonal antibody (#TA 150121 Origene, dilution of 1:200) over night. After washing, the abovementioned secondary antibodies against human IgG (Alexa488-coupled) and mouse IgG (Alexa594-coupled) were applied, and cell nuclei were stained with DAPI.

### DFS-70 ELISA

Antibodies against DFS-70 were detected with a qualitative anti-DFS-70 ELISA (DFS-70ELISA (IgG) EUROIMMUN, EA 159z-9601G) at room temperature using an automated system (EUROIMMUN Workstation equipped with an ELISA processing unit). The kit contained all necessary components, either ready-to-use or as stock solutions. Sera were diluted 1:200 and processed on an automated system strictly following the manufacturer’s instructions. Washing was followed by incubation with ready-to-use peroxidase-coupled rabbit anti-human IgG and incubation with substrate (TMB/H_2_O_2_) and stopping. Optical density was assessed automatically on the instrument and compared with that of a calibrator provided with the kit. Results above the calibrator reading were regarded as positive, and results below the calibrator reading were regarded as negative.

### HEp2 cell staining

HEp2 staining was performed strictly according to the protocol supplied with the kit on a QUANTA-Lyser 4000 QL4K (Inova/Werfen) using the NovaLite ANA Kit (Inova/Werfen, Ref: #704320). Sera were routinely diluted 1:80 and incubated on 12-well glass slides coated with HEp2 cells, which were already fixed and permeabilized by the manufacturer. Bound serum antibodies were detected with a ready-to-use solution of anti-human IgG (FITC) in conjunction with DAPI to enable automated focusing. Titrations were performed by geometrical serial dilutions of the serum samples. Titers and staining patterns were assessed on-screen via QUANTA-Link software (Inova/Werfen) on a calibrated monitor supported by visual inspection.

### Imaging, statistics, and data processing

Tissue-based and cell-based assays were imaged using widefield and confocal microscopy. Images were processed using ImageJ software (14). All statistical analyses were performed using Prism Version 9.4.1 (GraphPad Software, San Diego, CA). Two-sided Fisher’s exact tests were used for comparisons of categorical variables. p<0.05 indicated statistical significance. Data visualization was conducted in Prism Version 9.4.1 (GraphPad Software, San Diego, CA) and Inkscape Version 1.2.1 (Inkscape Project. 2020, available at https://inkscape.org).

### Ethics approval and consent to participate

The study was conducted in accordance with guidelines framed by the Declaration of Helsinki. All participants included in this study provided written informed consent for study participation and serum biomarker analyses. The Institutional Review Board of Charité Universitätsmedizin Berlin approved all the analyses included in the study (institutional review board #EA2/102/22).

## Results

### Characteristics of the study population

Clinical and paraclinical data of the study cohort have already been published. (9) In summary, the mean age of the PCVS patients was 41 years (range of 21–62 years), and 60% were females. All but one (98%) of the patients in the cohort had received an mRNA vaccine. Symptom onset after vaccination ranged from one hour to 30 days, with a median of three days. The majority of patients reported predominant symptoms of the peripheral nervous system, namely, paresthesia (n=28 (56%)), neuropathic pain (n=11 (22%)), fasciculations (n=11 (22%)), and myalgia (n=11 (22%)). Additionally, patients reported fatigue (n=23 (46%), cognitive deficits (n=18 (36%), and headaches (n=15 (30%)). Routine laboratory tests, electrophysiological assessments and skin biopsies did not reveal relevant pathological findings. Notably, screening for autoreactive antibodies showed positive ANA results in 11/45 patients (titer ranging from 1:160 to 1:640), positive ENA results in 2/46 patients, elevated cANCAs in 1/46, and no elevated pANCAs in 46 patients’ sera. The flowchart in **Supplementary Figure 1** depicts the number of patients included in the study’s assays.

### High frequency of IgG autoantibodies against PNS tissue in PCVS sera

**Figure 1:**
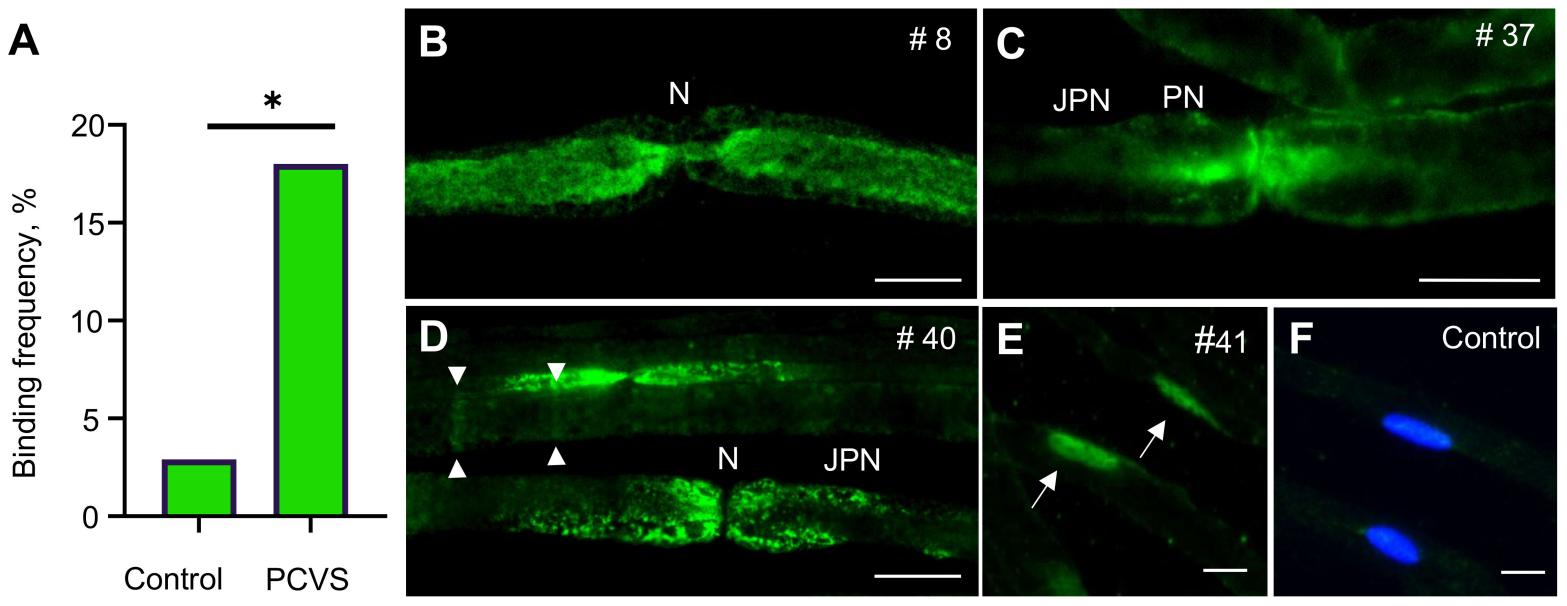
Frequency and patterns of IgG reactivity against peripheral nerve structures on sciatic nerve teased fibers. **(A)** Comparison of binding frequencies (% of total) in PCVS patients versus controls on mouse sciatic nerve teased fibers using the Fisher’s exact test; * indicates statistical significance at p<0.05; p=0.04. **(B)** Serum of patient #8 showing reactivity against axons. **(C)** Serum of patient #37 showing reactivity against paranodes (narrow). **(D)** Serum of patient #40 showing reactivity against paranodes (broad) and SLIs (arrowheads). **(E)** Serum of patient #41 showing reactivity against Schwann Cell nuclei (arrows). **(F)** Control serum showing no binding as absence of green fluorescence, cell nuclei are stained with DAPI. N, Node of Ranvier; PN, paranode; JPN, Juxtaparanode. Scale bar in B-D: 10 µm.

Testing sera for autoreactivity against peripheral nerve antigens, we identified binding to various structures of murine sciatic nerves in 9/50 (18%) patients, compared to only 1/35 control serum (3%) (p=0.04) (**Figure 1A** and **Table 1**). IgG-mediated binding in PCVS sera was directed against axons (n=4) (**Figure 1B**), and paranodes (n=5), including narrow (n=3) (**Figure 1C**) and broad paranodal binding (n=2) (**Figure 1D**). Further reactivity occurred against Schmidt-Lanterman incisures (SLIs) (n=2) (**Figure 1D**) as well as strongly against Schwann cell nuclei (n=1) (**Figure 1E**). One healthy control serum showed broad paranodal binding (data not shown), while 34/35 healthy controls and 41/50 patients remained without specific binding pattern (**Figure 1F**). Other frequently found antibodies against Schwann cell nuclei with low intensity in PCVS patients and healthy controls were considered unspecific. Testing for known autoantibody targets in peripheral nervous system tissue, 45/45 PCVS sera revealed no relevant IgM or IgG binding to gangliosides (**Supplementary Tables 1, 2**), indicating that gangliosides are not the underlying epitopes of the observed reactivity on peripheral nerves.

**Table 1:** Post COVID-19 vaccination syndrome patients with IgG reactivity against mouse sciatic nerve teased fibers.

Patient, age, sex	Main symptom(s)	Symptom onset	Staining pattern
#8, 61y, m	Paresthesia, myalgia	4 days	axon
#12, 41y, f	Paresthesia, fatigue	19 days	axon
#4, 40y, f	Neuropathic pain, paresthesia	hours	paranode (narrow)
#1, 47y, f	Cognitive impairment, fatigue	12 hours	axon and paranode (narrow)
#28 40y, m	Paresthesia, headache	7 days	paranode (broad), SLI
#50, 43y, f	Headache, paresthesia	1 day	axon
#37, 39y, m	Cognitive impairment, vertigo	10 days	paranode (narrow)
#40, 36y, f	Fatigue, cognitive impairment, paresthesia	10 days	paranode (broad), SLI
#41, 49y, f	Paresthesia, myalgia	9 days	Schwann cell nuclei

Patients’ age, sex, main symptoms, symptom onset after vaccination, and staining patterns are listed. y, years; m, male; f, female; SLI, Schmidt-Lanterman incisure.

### Autoantibody target identification revealed neurofilament subunits and DFS-70 as potential antigens

**Figure 2:**
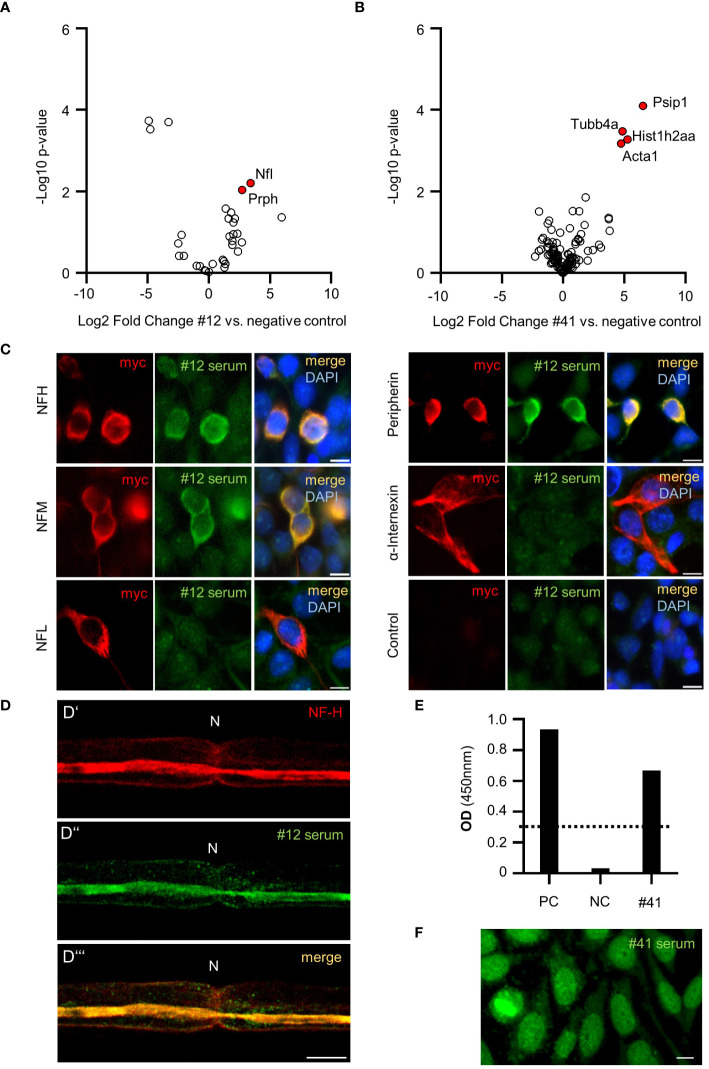
Antibody target identification and confirmation in PCVS sera. Volcano plot representing significantly enriched proteins (labeled in red) in patient #12 IgG IP **(A)** and patient #41 IgG IP **(B)** compared to a negative control; in A-B: the x-axis displays the log2-transformed fold change, and the y-axis represents the -log10-transformed p value. **(C)** Cell-based assays with patient #12 serum testing IgG reactivity against neurofilament subunits and control HEK293 cells. **(D)** Costaining of sciatic nerve teased fibers with a commercial NF-H antibody (D’) and patient #12 serum (D’’) showing clear signal overlap (D’’’). **(E)** ELISA analysis of DFS-70 and patient #41 serum. PC: positive control serum. NC: negative control serum. The standard reference serum OD was 0.278 (dotted line). OD: optical density. **(F)** Hep2 staining of patient #41 serum resembling fine speckled nuclear staining typical of DFS-70 IgGs.

To identify the unknown targets of these autoreactive IgGs, we performed IP-MS with nervous system tissue using the strongest binding sera against paranodes and SLIs (patient #40), axons (patient #12), and Schwann cell nuclei (patient #41). By comparing the IP-enriched proteins of patient #12 to those of a healthy control tissue nonbinder, we discovered the neurofilament subunits NF-L and PRPH as potential autoantibody targets (**Figure 2A**). In the serum of patient #41, Psip1 (protein name DFS-70) was identified as a potential target (**Figure 2B**) in accordance with the positive ANA screen and HEp-2 pattern AC-2, which is associated with the DFS-70 antigen. Notably, patient #41 additionally had positive cANCAs. Analysis of the third serum sample (patient #40) did not reveal any plausible protein hits (data not shown).

### Neurofilaments and DFS-70 were confirmed as autoantibody targets in cell-based assays and ELISA

The neurofilament subunits NFH, NFM, NFL, PRPH and the associated protein INA are well-known autoantibody targets (13). To investigate direct autoantibody binding in patient #12 serum, we performed cell-based assays in which these subunits were expressed separately in HEK293 cells. NF-H, NF-M, and PRPH-transfected cells exhibited clear binding to patient #12 serum, while NF-L and INA-transfected cells and the nontransfected control HEK293 cells did not show IgG reactivity (**Figure 2C**). Furthermore, costaining of patient’s #12 serum and a commercial NF-H antibody on sciatic nerve teased fibers showed clear signal overlap at the axon (**Figure 2D**). DFS-70 autoreactivity in the serum of patient #41 was confirmed in a commercial DFS-70 ELISA (**Figure 2E**). Additional validation experiments on HEp2 cells showed the expected fine speckled pattern (**Figure 2F**), similar to the pattern observed in Schwann cell nuclei (as shown in **Figure 1D**).

## Discussion

In this study, the serum prevalence of autoreactive IgGs against peripheral nerve structures was 18% in PCVS patients, which was statistically significantly higher than that in healthy controls. Binding occurred against axons, paranodes, SLIs, and Schwann cell nuclei. None of the PCVS sera bound to gangliosides. Subsequent target identification revealed various NF subunits as well as DFS-70 as autoantibody epitopes resembling the binding to axons and Schwann cell nuclei in two serum samples. However, the epitopes of autoreactive antibodies against paranodes and SLIs in a third serum sample could not be identified with our approach.

To our knowledge, this is the first report on autoreactive antibodies against peripheral nerve structures in patients suffering from neurological symptoms following COVID-19 vaccination offside reports on adverse events such as ganglioside antibody-positive Guillain–Barré syndrome (15). The concept of autoreactive antibodies targeting proteins in nodes and paranodes as effectors of severe inflammatory peripheral neuropathies, as well as their detection with indirect immunofluorescence on murine teased fibers is well established (16). Furthermore, CSF autoantibodies against yet undefined brain epitopes were associated with worse cognitive performance in PCS patients (17). Whether autoantibodies detected in PCS patients differ from those detected in PCVS is not known to date. The evidence supporting the pathophysiological relevance of serum autoantibodies against nervous system tissues, beyond the encephalitis spectrum, is expanding. This trend is based on the increasing number of studies showing links between autoantibody serostatus and unfavorable clinical outcomes in stroke (18), dementia (19), and cancer (20) patients.

The common lack of functional investigations in these and our studies, however, does not allow conclusions on the direct pathogenicity of those autoantibodies directed against brain and nerve epitopes. In fact, the significance of serum autoantibodies targeting NFs detected via Western Blots, ELISA and cell-based assays without considering tissue reactivity has been previously questioned (13). Moreover, the sole detection of DFS-70 antibodies is considered to be a negative predictor of autoimmune rheumatic diseases associated with ANA (21, 22). Conversely, other studies have shown the pathological effects of serum NF-H antibodies ex vivo (23) and DFS-70 antibodies *in vitro* (24) as well as the diagnostic value of both NF antibodies in various neurological diseases (25) and DFS-70 antibodies in atopic dermatitis (26). In particular, CSF antibodies targeting NFs were reported to be biomarkers of axonopathies in inflammatory neurological disorders if they were detected in both tissue-based and cell-based assays at a titer of 1:600 or higher in the latter (27). In general, the here-identified autoantibodies targeting peripheral nerve structures – if considered pathogenic – could have different mechanisms of actions such as complement-dependent and antibody-dependent cellular cytotoxicity, antibody-mediated crosslinking and antigen internalization, as well as disruption of protein-protein interactions as previously described for anti-neuronal autoantibodies (28).

Due to the wide range of symptom onset after vaccination in our cohort (one hour to 30 days), the heterogenous clinical presentation, and the single cross-sectional serum investigation, we can speculate only about the origin of these autoantibodies and their relation to the vaccine. They could be present prior to vaccination and become relevant upon vaccine-induced autoimmunity, as discussed for autoimmune/inflammatory syndrome induced by adjuvants (ASIA) (29). Alternatively, they might have been induced by the vaccination itself, as proposed for anti-idiotype antibodies and platelet-activating antibodies (11, 30). Here, several mechanisms for antibody formation such as molecular mimicry, the formation of immune complexes with vaccine components as well as unspecific B-cell activation and subsequent expansion in a pro-inflammatory milieu could be relevant. The discovery of these antibodies as innocent bystanders of a heterogeneous disease spectrum also seems plausible, as autoantibodies in general are present in healthy individuals (31, 32). Future investigations of patient sera prior to and after vaccination, as well as functional tests with patient-derived antibodies, are needed to provide a mechanistic understanding of autoantibody origin and pathophysiological relevance.

Considering the total number of vaccinated people, PCVS still seems to be rare, and the overall severity of symptoms in PCVS patients appears by far less critical than well-defined neurological complications after vaccination (33). However, until now, there are neither diagnostic criteria nor biomarkers available for facilitating the diagnosis of PCVS, and distinguishing PCVS from primarily psychiatric or psychosomatic diseases has been very challenging (9). Given that our clinical findings have only recently been confirmed in two other PCVS cohorts (34), we propose to further investigate the biomarker potential and pathophysiological role of autoantibody reactivity in larger groups of PCVS patients.

## Data availability statement

The raw data supporting the conclusions of this article will be made available upon reasonable request.

## Ethics statement

The studies involving humans were approved by the Institutional Review Board of Charité Universitätsmedizin Berlin (#EA2/102/22). The studies were conducted in accordance with the local legislation and institutional requirements. The participants provided their written informed consent to participate in this study. The animal study was approved by the Animal Welfare Office at Charité Universitätsmedizin Berlin (#T-CH-0009/22). The study was conducted in accordance with the local legislation and institutional requirements. Written informed consent was obtained from the individual(s) for the publication of any potentially identifiable images or data included in this article.

## Author contributions

FA: Conceptualization, Data curation, Formal analysis, Investigation, Methodology, Visualization, Writing – original draft, Writing – review & editing. AB: Conceptualization, Data curation, Formal analysis, Investigation, Methodology, Writing – original draft, Writing – review & editing. ET: Investigation, Writing – review & editing. FB: Investigation, Writing – review & editing. MK: Data curation, Formal analysis, Investigation, Visualization, Writing – review & editing. PM: Investigation, Writing – review & editing. ES-S: Investigation, Writing – review & editing. MN: Investigation, Writing – review & editing. MM: Investigation, Writing – review & editing. MB: Investigation, Writing – review & editing. ME: Funding acquisition, Investigation, Writing – review & editing. HP: Conceptualization, Funding acquisition, Investigation, Methodology, Resources, Supervision, Writing – original draft, Writing – review & editing. CF: Conceptualization, Investigation, Methodology, Resources, Supervision, Writing – original draft, Writing – review & editing.
